# Host evolutionary relationships explain tree mortality caused by a generalist pest–pathogen complex

**DOI:** 10.1111/eva.13182

**Published:** 2021-01-05

**Authors:** Shannon Colleen Lynch, Akif Eskalen, Gregory S. Gilbert

**Affiliations:** ^1^ Department of Environmental Studies University of California Santa Cruz Santa Cruz California USA; ^2^ Department of Plant Pathology University of California Davis Davis California USA

**Keywords:** biological invasions, *Euwallacea*, Fusarium dieback, host specificity, infectious diseases, invasive plant pests, invasive shot hole borers, phylogenetic signal

## Abstract

The phylogenetic signal of transmissibility (competence) and attack severity among hosts of generalist pests is poorly understood. In this study, we examined the phylogenetic effects on hosts differentially affected by an emergent generalist beetle–pathogen complex in California and South Africa. Host types (non‐competent, competent and killed‐competent) are based on nested types of outcomes of interactions between host plants, the beetles and the fungal pathogens. Phylogenetic dispersion analysis of each host type revealed that the phylogenetic preferences of beetle attack and fungal growth were a nonrandom subset of all available tree and shrub species. Competent hosts were phylogenetically narrower by 62 Myr than the set of all potential hosts, and those with devastating impacts were the most constrained by 107 Myr. Our results show a strong phylogenetic signal in the relative effects of a generalist pest–pathogen complex on host species, demonstrating that the strength of multi‐host pest impacts in plants can be predicted by host evolutionary relationships. This study presents a unifying theoretical approach to identifying likely disease outcomes across multiple host‐pest combinations.

## 1 INTRODUCTION

Accidental introductions of plant pests (e.g., fungi, bacteria, viruses, animals, plants) into areas outside their place of origin have resulted in novel species interactions that pose ecological and economic threats to agricultural, urban and wildland landscapes (Donatelli et al., 2017; Goodell et al., 2000; Parker & Hay, 2005; Pimentel et al., 2000; Young et al., 2017). To respond appropriately to such threats and optimize the use of limited resources for management, decision‐makers require robust analytical tools that help determine in which plant communities emergent pests are most likely to establish and cause damage during critical early stages of invasions. As a necessary first step to developing predictive models of pest spread in novel habitats, we take an evolutionary ecology approach and examine how the host range structure of different pest–pathogen combinations can be used to better understand mechanisms of their establishment, spread and impacts.

Evolutionary tools show promise as a way to understand invasions and predict host range of pests in novel locations (Briese, 2003; Fountain‐Jones et al., 2018; Gilbert et al., 2012). For plants and their pathogens, evolutionary constraints in physiological, morphological and chemical traits that confer host susceptibility or pathogen virulence produce a phylogenetic signal for host range; hence, closely related plants are more likely to share pests and pathogens (Gilbert & Webb, 2007; Young et al., 2017). Phylogenetic signal in host range has been used to predict the likely host range of generalist plant pests in local communities not yet invaded by such pests (Gilbert & Parker, 2016; Parker et al., 2015). Patterns of phylogenetic signal in host range have been well documented for plant–pest relationships involving a single pest interacting with their host plants (e.g., plant–pathogen, plant–insect), but not for those exhibiting multiple interactions (e.g., pest–pathogen complexes) where the traits shaping the relationships may differ among the multiple partners and their interactions. As such, the patterns and strength of the signal as a basis for risk analysis for more complex plant–pest problems are less well understood. Here, we use an emergent invasive pest–pathogen complex affecting a diversity of tree hosts in Southern California to test the utility of this phylogenetic tool in evaluating host range for novel plant–insect–pathogen interactions. Further, we assess whether we can use information on the phylogenetic structure of the pest–pathogen host range in California, where the complex has been intensively studied, to guide an understanding of likely patterns in South Africa and inform priorities for phytosanitary surveillance, where the invaders have only recently established.

**FIGURE 1:**
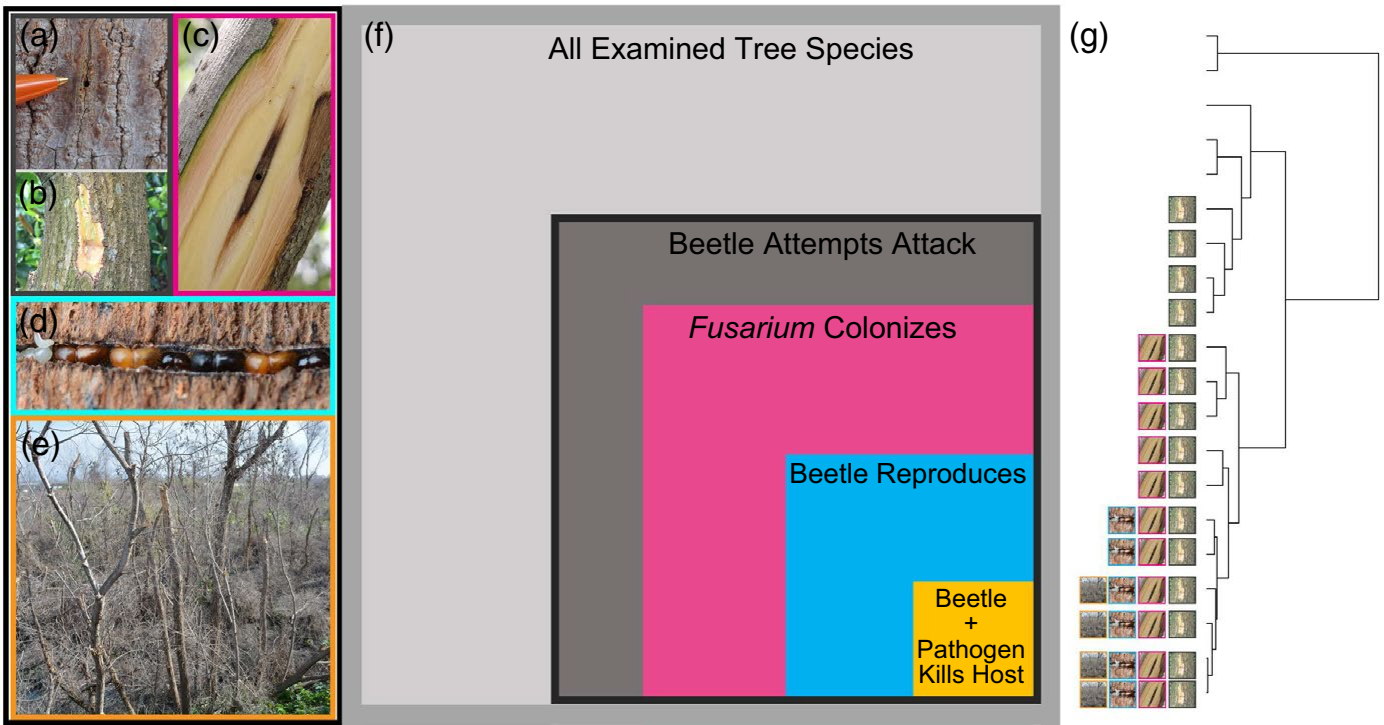
Representation of the expected phylogenetic effects on different host types impacted by Fusarium dieback‐invasive shot hole borers. The left panel (a‐e) depicts examples of nested types of outcomes of interactions between host plants, the beetles, and the fungi. **Non‐competent** **hosts** (a‐c) represent tree species that do not support beetle reproduction or fungal transmission. For host types on which the beetle **attempts an attack** (a‐b), entry holes are observed but removal of the bark reveals healthy tissue and no signs of a gallery. Removal of the outer bark on hosts susceptible to ***Fusarium*** **colonization** (c) reveals necrotic tissue caused by the pathogen, but no signs of a gallery. On **competent hosts** (d), the beetle is able to establish a natal gallery and produce offspring and on some of these (e), the beetle and pathogen can kill the host (i.e., **killed‐competent**). Successfully established breeding galleries in competent hosts contain a “fungal garden” and beetles at all life stages (eggs, developing larvae, adults), demonstrating the beetles’ ability to cultivate their nutritional symbiotic fungi and complete their life cycle. Colours around each image correspond to the host type represented by the nested boxes in the middle panel (f), the sizes of each which correspond to the relative proportion of tree species for each host type. The phylogenetic tree in the right panel (g) depicts our hypothesis that hosts are a nonrandom, closely related, subset of all available tree species and that this phylogenetic signal is more pronounced for each of the nested interaction outcomes. The icons represent the examples of the nested types of interaction outcomes from most inclusive to least inclusive

Fusarium dieback–invasive shot hole borers (FD‐ISHB) is a pest–pathogen complex with a broad host range that involves two cryptic ambrosia beetles (PSHB & KSHB, Table 1) in the *Euwallacea* species complex (Coleoptera: Curculionidae: Scolytinae; Gomez et al., 2018; Smith et al., 2019; Stouthamer et al., 2017) and the specific symbiotic fungal pathogens each beetle species carries (Table 1 and S1; Freeman et al., 2013; Lynch et al., 2016; Na et al., 2018). The beetles were introduced to California from Southeast Asia (Eskalen et al., 2012; Stouthamer et al., 2017), presumably on packing material. Since the appearance of ISHB in California in 2012, the combined effects of ISHB and their fusaria symbionts have killed or caused dieback on 77 tree species on which the beetles can reproduce, but the beetles make attempted attacks on an additional 247 tree species (Figure 1, Table S1; Eskalen et al., 2013). The two pest–pathogen complexes that form FD‐ISHB have indistinguishable host ranges. Critically, the recent introduction of one of those complexes to South Africa, the polyphagous shot hole borer (PSHB, Table 1; Paap et al., 2018a) has been cause for concern given the severe damage these invasive species have caused in California. The known host range in California and South Africa continues to grow, pointing to the need for a sound scientific understanding of the complexity of the FD‐ISHB host range to inform risk assessments and focus phytosanitary actions in areas where the beetles have established, and in noninvaded locations worldwide that have favourable conditions for their establishment.

**TABLE 1:** Insect vectors and corresponding fungal pathogens causing Fusarium dieback on tree hosts in California, Israel, and South Africa

Invasive shot hole borers		Year detected/Established	Fusaria pathogens	Other weak mycangial pathogens	
Species name	Common name				
*Euwallacea fornicatus* ^a^, ^b^	Polyphagous shot hole borer (PSHB)	Israel: 2005^c^ CA: 2003/2012 ZA: 2016	*Fusarium euwallaceae* ^d^	*Graphium euwallaceae* ^e^	*Paracremonium pembeum* ^e^
*Euwallacea kuroshio* ^a^	Kuroshio shot hole borer (KSHB)	CA: 2014	*Fusarium kuroshium* ^f^	*Graphium kuroshium* ^f^	

^a^ Gomez et al. (2018).

^b^ Smith et al. (2019).

^c^ Mendel et al. (2012).

^d^ Freeman et al. (2013).

^e^ Lynch et al. (2016).

^f^ Na et al. (2018).

While a large body of work has established there is a phylogenetic signal in overall host ranges of pests and pathogens (Gilbert & Parker, 2016), the phylogenetic signal of competence and severity among hosts is much less well understood (Gilbert et al., 2015). In addition to distinguishing between hosts that do not support reproduction of the beetle‐pathogen (non‐competent) and those that do (competent), phylogenetic relatedness may also predict those hosts that are killed by the beetle‐pathogen (killed‐competent; Figure 1). For FD‐ISHB, different host types (non‐competent, competent, and killed‐competent) are based on nested types of outcomes of interactions between host plants, the beetles and the fungi (Figure 1). Hosts that are competent for pest reproduction are the most important in driving the spread of invasive enemies, and the lethality to different hosts is the most important for ecological impact. Thus, assessing the phylogenetic signal of host competency is key to evaluating the potential for establishment, spread and damage from novel pests and pathogens.

The apparent damage caused by complex novel pest invasions such as FD‐ISHB highlights the need to strategically apply, in early response efforts, an understanding of the phylogenetic signal in competence and severity among their hosts. The 77 currently recognized competent host species occur across varied and complex landscapes, with important implications for the ecological and economic vitality of a variety of systems. For example, the California avocado industry, which produces 90% of the United States domestic crop, has spent over $2.5 million to combat the problem. For urban forests, initial estimates suggest that FD‐ISHB has the potential to kill roughly 27 million trees (38%) in Southern California's 10,992‐square kilometre urban region (McPherson et al., 2016). In Orange County, California, the removal of 1524 infested trees and treatment of 2228 trees cost the county approximately $3 million between 2013 and 2017 (Parks, 2017). Costly large‐scale tree removal efforts to manage the problem could have unintended consequences for the environment and public health, given that urban forest trees in California remove 567,748 t CO_2_ annually, equivalent to the annual output of 120,000 cars (McPherson et al., 2016). FD‐ISHB has also resulted in the loss of hundreds of thousands of trees in riparian ecosystems of Southern California (Boland, 2016; Parks, 2017), habitat critical for breeding by endangered bird species and highly vulnerable to encroachment of damaging invasive plant species.

In South Africa, the PSHB infestation is currently in a stage similar to the situation in California in 2012. At that time, the beetle was discovered in the Los Angeles basin on a backyard avocado tree but had not yet established in commercial groves, and the damage it caused was restricted to urban forests and botanical gardens (van den Berg et al., 2019; Eskalen et al., 2012). A rapid monitoring response uncovered the broad host range of the pest–pathogen complex (Eskalen et al., 2013), but its ability to establish in native vegetation was only gradually recognized. Similarly, in South Africa today the most visible impact of the PSHB invasion is in urban forests, and the beetle has not yet been detected in commercial avocado groves (https://www.fabinet.up.ac.za/pshb). Given that wildland habitats differ in vegetation composition in California and South Africa, the impact of the invasion on South African native forests is unclear. Reports of the beetle occurring in eight of the nine provinces in South Africa and spreading from urban areas into native forests suggest those habitats are invadable (https://www.fabinet.up.ac.za/pshb). However, which species will be affected, and to what extent, is unknown. Understanding the influence of host range on FD‐ISHB impacts during this key phase of the infestation in South Africa is therefore imperative.

In this study, we tested the hypothesis that hosts supporting ISHB‐*Fusarium* reproduction are more strongly phylogenetically constrained than non‐competent hosts. As such, we expect that the probability of finding ISHB on two host species declines with phylogenetic distance between the hosts, and this decline is steeper for competent hosts. Moreover, we expect that phylogenetic signal in host range is stronger on competent hosts that are killed when attacked.

## 2 METHODS

### 2.1 Host range assessment

The FD‐ISHB host range comprises 77 host species that support beetle reproduction (competent hosts), 18 of which are killed when attacked **(**Figure 1, Table S1). The adult beetles make attempted attacks on another 247 species in 61 families that do not support their reproduction (non‐competent hosts), although the fungi can colonize and cause necrosis on 137 of these non‐competent hosts (Figure 1, Table S1; Eskalen et al., 2013). These non‐competent hosts are never killed when attacked. The specific definitions and details for each of these categories are provided in Figure 1. The host range in California was determined in a previous study of heavily infested botanical gardens at the epicentre of the infestation in Los Angeles County (Eskalen et al., 2013), and subsequent systematic surveys of 23,588 trees from 2012 to 2019 in a variety of habitats throughout San Diego, Orange, Los Angeles, San Bernardino, Ventura, Santa Barbara, Riverside and San Luis Obispo Counties (Lynch *in prep*; https://ucanr.edu/sites/pshb/Map). The botanical gardens harbour a wide range of plant species that represent unique and common ecosystems worldwide and contain all the host species that occur throughout urban and wildland forests in Southern California. Seven competent and 25 non‐competent hosts were similarly identified in a separate survey of the national botanical gardens of South Africa through the International Plant Sentinel Network tree health monitoring program (Paap et al., 2018a, 2018b) and preliminary surveys of national nature reserves and urban forests throughout all nine provinces in 2017–2019 (Wilhelm de Beer, *personal communication*; https://www.fabinet.up.ac.za/pshb; Table S1). In California, surveys were conducted by trained experts representing the University of California (UC) Riverside, Santa Cruz, and Davis; UC Cooperative Extension; Orange, San Diego, Los Angeles and Ventura County Agriculture; USDA Forest Service, Forest Health Protection; California Department of Forestry and Fire Protection; Disney; the Huntington Library Art Collections and Botanical Gardens; and the Los Angeles County Arboretum and Botanic Gardens. Experts conducting surveys in South Africa represent the Forestry and Agricultural Biotechnology Institute (FABI) at the University of Pretoria; Stellenbosch University; Rhodes University; South African National Biodiversity Institute; and the City of Johannesburg Metropolitan Municipality.

For each individual tree, surveyors recorded at minimum the tree location, species, and the presence or absence of FD‐ISHB based on the unique symptoms caused by the beetles and fungi as described in Eskalen et al. (2013). Tree species not exhibiting FD‐ISHB symptoms, but in areas with active infestations, were classified as apparent nonhosts. In all cases of new tree species exhibiting symptoms characteristic of FD‐ISHB, fungal and beetle identities were confirmed using morphological and molecular identification techniques described in Eskalen et al. (2013). Suitability for reproduction was confirmed by the presence of eggs, larvae, pupae or teneral females, or by the presence of males in the galleries of infested trees.

### 2.2 Analyses

To estimate the time of independent evolution between plant species (phylogenetic distance), we first created a hypothesis for the phylogenetic relationships among tree and shrub species in California and South Africa using the R2G2_20140601 supertree of; see Data S1 for newick file). This tree includes dated nodes for all angiosperm families given by the Angiosperm Phylogeny Group classification III (APG III; Bremer et al., 2009) as well as gymnosperm and monilophyte families; the tree was dated using Wikström ages (Davies et al., 2004; Wikström et al., 2001) and additional consensus dates from the literature, with all nodes in the tree given stable dates (Parker et al., 2015). We used this tree rather than basing our phylogenetic tree on APG IV (Byng et al., 2016) to be consistent with and comparable to the validated work on phylogenetic signal in host ranges in the previous studies. All 2717 taxa for which the beetles could encounter in California or South Africa include native and non‐native trees and shrubs found across agricultural, urban and wildland landscapes, and were compiled using the CalFlora, West Coast Arborists, The Plant List, and Dendrological Society of South Africa curated databases (Data S1). We used Phylomatic version included in Phylocom v4.2 (Webb et al., 2008) to create a pruned ultrametric tree of all genera in the database, with branch lengths that reflected the estimated time between branching events (Data S1).

In the absence of information about intrafamilial phylogenetic resolution, relationships from the R2G2_20140601 supertree are modelled as polytomies. To improve estimates of phylogenetic signal between hosts exhibiting different levels of attack, we reviewed the literature to resolve polytomies across taxa that interacted with the beetle and/or the *Fusarium* pathogens. Taxa comprised genera in the Fabaceae including *Acacia* (Gómez‐Acevedo et al., 2010; Kyalangalilwa et al., 2013; Miller et al., 2011; Miller & Seigler, 2012), *Senegalia* (Kyalangalilwa et al., 2013), *Vachellia* (Kyalangalilwa et al., 2013), *Prosopis* (Catalano et al., 2008), *Erythrina* (Bruneau, 1996; De Luca et al., 2018) and *Bauhinia* (Hao et al., 2003; Meng et al., 2014; Sinou et al., 2009); genera in the Lauraceae including *Cinnamomum*, *Cryptocarya* (Chanderbali et al., 2001); and genera in the Salicaceae, including *Salix* and *Populus* (Hamzeh & Dayanandan, 2004; Lauron‐Moreau et al., 2015; Liu et al., 2016; Wang et al., 2014; Zhang et al., 2018; Zhou et al., 2018). Topologies for *Acer* (Grimm et al., 2007; Harris et al., 2017; Li et al., 2006, 2019; Suh et al., 2000; Tian et al., 2002), *Platanus* (Feng et al., 2005; Grimm & Denk, 2008) and *Quercus* (Cavender‐Bares & González‐Rodríguez, 2015; Hipp et al., 2014, 2018; Manos et al., 1999, 2001) were additionally resolved. Finer scale node ages were then estimated by interpolation using the Phylocom bladj function in Phylomatic v4.2 (Webb et al., 2008). From this finer resolution tree, we used the phydist function in the R package Picante v. 1.2–0 (Kembel et al., 2010) to calculate pairwise phylogenetic distances for each pair of plant species, which is twice the time to the most recent common ancestor in Myr. The case of zero phylogenetic distance (distance from a known host species to itself) was included in the analysis.

We performed a phylogenetic dispersion analysis of phylogenetic distances for all examined tree species, confirmed nonhosts, non‐competent hosts (attempted host attack only and attacked hosts suitable for fungal colonization), and all competent host species and their subsets of those that are killed or not killed when attacked. We followed approaches used in previous publications and inspected the cumulative distribution of phylogenetic distances between species pairs (CDPD), which provides useful information on the depth of trait conservatism in plant‐pathogen interactions (Gilbert & Parker, 2016; Parker et al., 2015). Overlap of CDPD curves between all examined tree species and host tree species indicates that hosts are a random subset of all available tree species (no phylogenetic signal). A downward shift in the host CDPD curve indicates that host species are a more closely related subset of all available tree species than expected at random, because the removal of more distantly related clades retains shorter distances (phylogenetic signal). We expect these downward shifts to be more dramatic with hosts that are increasingly more severely impacted by the beetle‐fungal interactions. Measures of mean phylogenetic distance in pest host ranges across broad plant phylogenies tend to be dominated by the influence of many long phylogenetic distance pairings (Gilbert & Parker, 2016). Additionally, nearest phylogenetic distance measures can be unstable because they do not reflect the plant community as a whole. In addition to examining the overall CDPD, we follow Parker et al. (2015) and compare distances at the 10th quantile, which were found to be more informative than mean distances for plant–fungal interactions because it reduces the structural swamping effect of many distantly related pairs in phylogenies.

In addition to phylogenetic dispersion analysis, we measured the strength of the phylogenetic signal (*D*) for binary traits using the phylo.d function in the R package caper v.1.0.1. This measure developed by Fritz and Purvis (2010) is computed by scaling the observed sum of sister‐clade differences in a given phylogeny with the mean values of simulated expected distributions under Brownian motion and a random phylogenetic pattern. The given *D* statistic is scaled between 0 and 1, where a value of 1 indicates phylogenetic randomness. All analyses were performed using R statistical framework, with functions from the Picante v. 1.2–0, Vegan v. 1.17–8, Hmisc v. 4.3.0, phytools v. 0.6, phangorn v. 2.5.5, Geiger v. 2.0.6.2, caper v. 1.0.1 and Stats v. 2.12.2 packages (http://cran.r‐project.org/).

## 3 RESULTS

### 3.1 Phylogenetic patterns of host‐pest interactions

**FIGURE 2:**
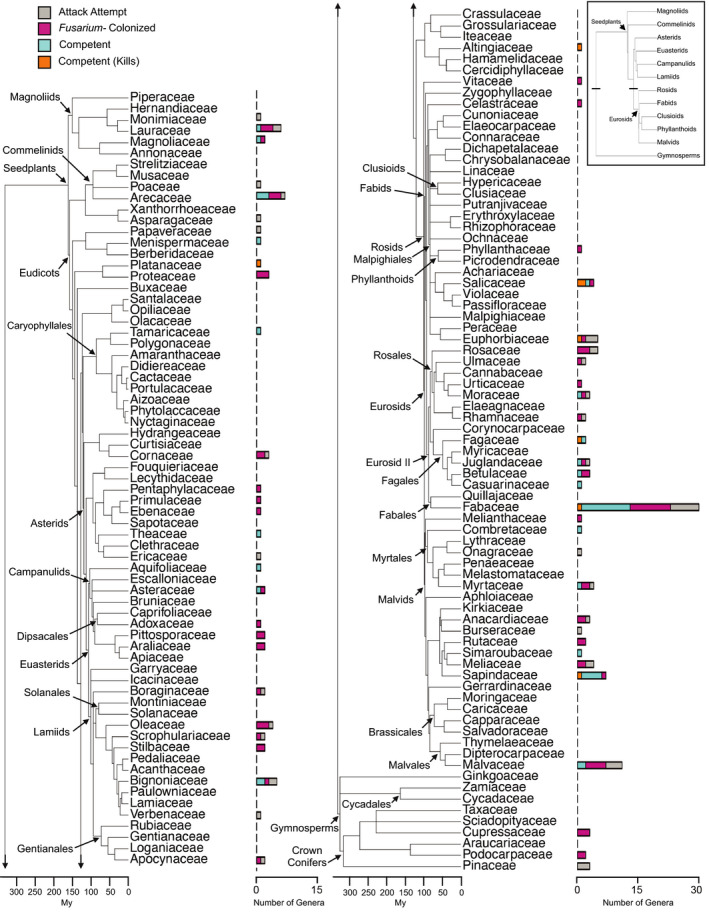
Phylogenetic tree of families representing all examined tree species in the present study. Stacked columns at the tree tips depict the nested types of outcomes of interactions between host plants, beetles, and fungi for genera within each family. Segments within each column represent the number of attacked genera with tree species that are *Fusarium*‐colonized, competent, and killed‐competent hosts within each family

The distribution of non‐competent and competent hosts exhibited a nested pattern across the phylogeny of potential host species in California and South Africa. Species that were attacked by the beetles clustered within 62 families and 170 genera within our geographic ranges (Figure 2). These taxa cover the range of angiosperm and some gymnosperm tree species. For gymnosperms, beetle attack attempts occurred on species within the “crown conifer” clade (Cupressaceae, Podocarpaceae, Pinaceae) but not species within other more distantly related groups (e.g., Ginkgoaceae or Cycadales; Figure 2). Other groups containing species free from beetle attack included families within the Caryophyllales (with the exception of Tamaricaceae), Malpighiales (with the exception of Phyllanthaceae, Salicaceae and Euphorbiaceae), and families within groups containing Huertales (Gerrardinaceae), Brassicales and Malvales (with the exception of Malvaceae; Figure 2). The beetles’ fusaria symbionts could colonize on a subset of 50 families and 122 genera of beetle‐attacked species across the phylogeny, including species within Cupressaceae and Podocarpaceae (Figure 2). The 77 competent host species clumped within 24 families and 48 genera of all attacked species. These species were nested within angiosperm lineages ranging from the most basal Magnoliids that diversified ~150 Mya to lineages that originated as recently as ~35 Mya (e.g., Malvaceae). Notably, 59 of the 77 competent host species (77%) and 14 of the 18 killed‐competent host species (78%) clustered within the Rosids clade (Figure 2). Within the Rosids, 43 competent (56%) and ten killed‐competent (55%) host species grouped within the Fabids; half of the competent host species were further clustered within the Eurosid II clade (Figure 2). Only killed‐competent hosts exhibited a significant phylogenetic signal measured by the *D* statistic (*D* = 0.299) and the strength of the signal indicated a clumped phylogenetic pattern consistent with Brownian motion (Table 2).

**TABLE 2:** Phylogenetic signal for each host type measured by *D* statistic, and the probability of E(D) resulting from Brownian phylogenetic structure

Host type	*D*	Probability of E(D)
Nonhost	0.8410635	0
Beetle only attacked	0.7404623	0
Fungus	0.7633496	0
Competent	0.7945735	0
Competent not killed	0.9098142	0
Competent killed	0.2993492	0.303

### 3.2 Phylogenetic dispersion analysis

**FIGURE 3:**
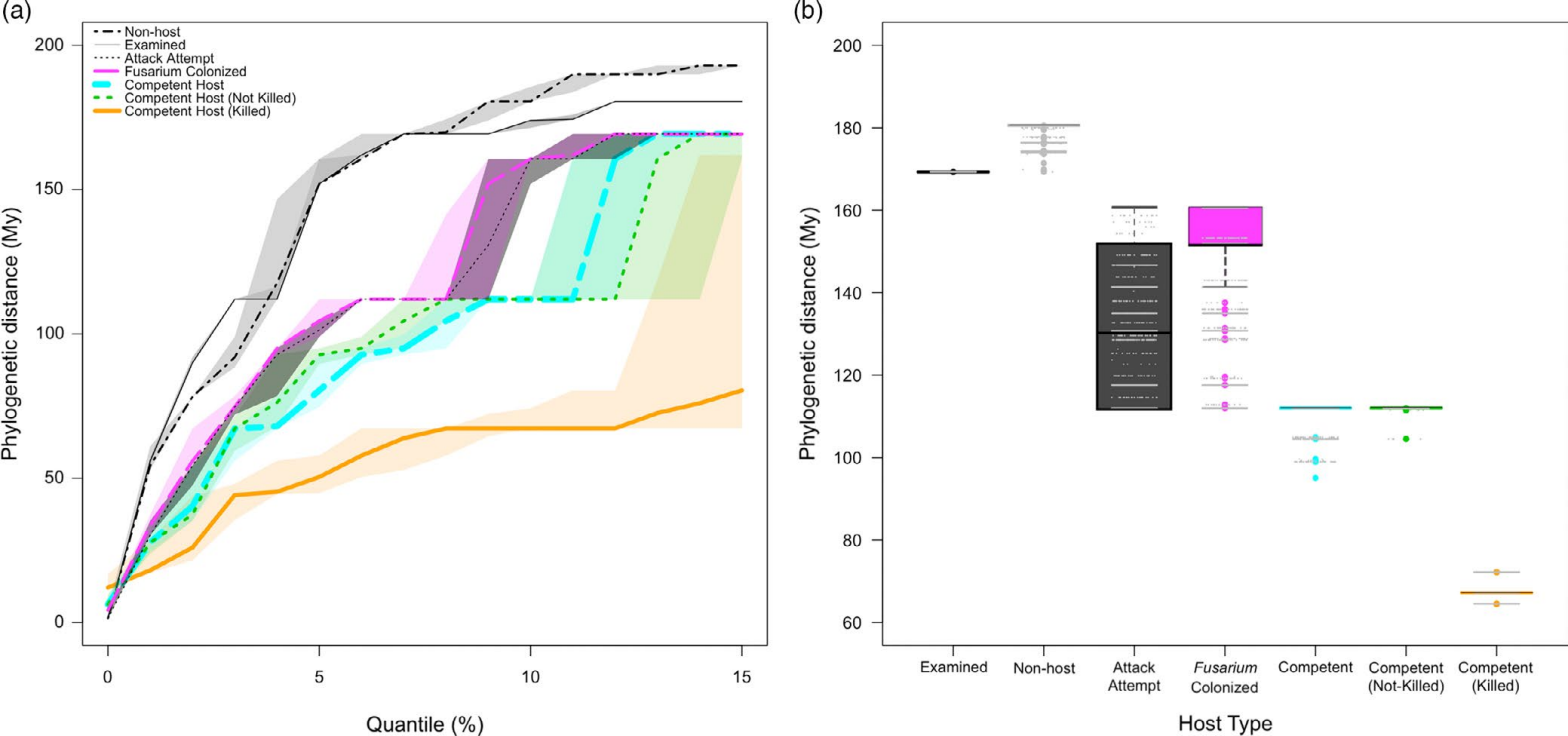
Phylogenetic distances for all species pairs of each host type (a‐b). Intervals represent the 95% confidence interval envelope generated from 10,000 bootstrap simulations on a random sample of 90% of the species within each host type. (a) Cumulative distribution of phylogenetic distances (CDPD) from quantiles 1%–15%. (b) Boxplots of phylogenetic distances at the 10th quantile. Grey dots represent actual data from the simulations

The phylogenetic distances for all pairs of the 2717 observed tree species and confirmed nonhosts from California and South Africa ranged between 1.4 and 806 Myr (Figure S1). This range decreased notably with increasingly severe nested types of outcomes of interactions between host plants, the beetles, and the fungus (Figure S1). We ranked the phylogenetic distances for all species pairs and their respective subsets (Figure 3a and S2,S3). Consistent with results in Parker et al. (2015), inspection of the full CDPD curves indicated that affected phylogenetic distances tend to be much shorter than the overall median because of the swamping effect of many distantly related pairs (Figure S2). As such, we focused our analysis at the scale of the 10th quantile of pairwise phylogenetic distances between species, where the depth of conservatism of important traits that confer host susceptibility is most informative (Figure 3b). As phylogenetic distance represents time of independent evolution (Myr), shorter distances indicate species are more closely related to one another.

Species that were attacked by beetles were a nonrandom subset of all the available hosts as indicated by a downward shift in their CDPD curve; the phylogenetic distances among the attacked hosts are consistently much shorter than those among all available species (Figure 3b). Shorter distances indicate a selectivity where if one species of tree is attacked, close relatives are also more likely to be attacked. The CDPD curves for beetle‐attacked and *Fusarium*‐colonized hosts overlapped, suggesting that the phylogenetic preferences for beetle attack and fungal growth are very similar. Notably, within those attacked hosts, an even more phylogenetically restricted subset of hosts was able to serve as competent hosts for beetle reproduction. A very striking phylogenetic effect was seen on the most severely affected competent hosts. Competent host species that were killed by beetle/fungal attack fell into phylogenetic clusters that produced a much flatter CDPD. Consistent with entire clades being lost from the host range with increasingly more severe interactions, these hosts for which attack was lethal had a decile phylogenetic distance of only 60 Myr, compared with 160 Myr for all the hosts attacked by the beetles (i.e., killed host species are much more closely related to each other than are all the species attacked by the beetles). Removal of gymnosperms from the host data revealed a shift in the CDPD for non‐competent hosts, but distances were still longer than competent hosts (Figure S3). Patterns were not different when South African trees were removed from the analysis (Figure S3).

## 4 DISCUSSION

In this study, we quantified the degree of phylogenetic signal in the host range of a new invasive generalist pest and pathogen complex from southeast Asia that elicits different effects across different host tree species. As we expected, the 327 tree species attacked by Fusarium dieback‐invasive shot hole borers (FD‐ISHB) in California and South Africa were phylogenetically constrained compared to all examined tree and shrub species. Additionally, competent hosts (those that support beetle reproduction) were more phylogenetically constrained than non‐competent hosts. Finally, those competent hosts that are killed when attacked exhibited the strongest phylogenetic signal. Phylogenetic dispersion analysis of each host type from the most inclusive (beetle attempts an attack) to most restrictive (beetle and pathogen kill their host) revealed that the phylogenetic preferences of beetle attack and fungal growth were the same, nonrandom subset of all available tree and shrub species. Competent host range was phylogenetically narrower than attacked hosts by 62 Myr, and those with devastating impacts were the most constrained, narrower by 107 Myr. As such, our results show a strong phylogenetic signal in the relative effects of FD‐ISHB on host species, demonstrating that the strength of multi‐host pest impacts in plants can be predicted by host evolutionary relationships. These findings form the basis for developing predictive models of multi‐host pest spread in novel habitats using tools in phylogenetic ecology.

### 4.1 Estimations of phylogenetic signal

Both phylogenetic dispersion analysis and the *D* statistical measure of phylogenetic signal (Fritz & Purvis, 2010) detected a phylogenetic effect on the most severely affected competent hosts. Phylogenetic dispersion analysis was potentially more sensitive in detecting a signal for non‐competent and all competent hosts than *D* because while there are “jumps” in the signal (i.e., roughly 25% of competent hosts occur outside the Rosids), we see high clustering within groups containing competent host species. Within the Rosids, there is another jump in the signal between the Fabids and Malvids, but a high degree of clustering occurs within those two groups, particularly in the Fabids (i.e., Salicaceae, Fagaceae and Fabaceae) and the Malvids (i.e., Sapindaceae). The *D* measure in phylogenetic signal is based on an underlying threshold model, which assumes that patterns of a binary trait across the phylogeny are based on one or more evolved, continuous traits (Fritz & Purvis, 2010). However, although many traits important in plant‐enemy interactions show a phylogenetic signal (Agrawal, 2007; Boller & Felix, 2009; Gilbert & Parker, 2016; Pearse & Hipp, 2009), there are exceptions (Becerra, 1997; Pichersky & Lewinsohn, 2011; Wink, 2003). Thus, our results suggest there are many ways for hosts to be susceptible. Those ways are moderately constrained phylogenetically, but susceptibility clusters within phylogenetic groups and this clumping becomes more restricted with more impactful interactions.

### 4.2 Phylogenetic signal in multi‐host pest interactions

Quantitative measures that leverage an understanding of the evolutionary ecology of host‐pest interactions to assess the relative impacts of generalist pests on their hosts provide important and novel tools to predict threats to ecosystems. By utilizing multiple invasion pathways, multi‐host pests present inherently different epidemiological dynamics than single host pests when introduced to naïve plant or animal communities. In particular, generalist pests do not rely on density‐dependent transmission of a single host species, which thereby increases the likelihood of pest‐induced host extinction (De Castro & Bolker, 2005; Smith et al., 2006). As the majority of plant and animal pests attack multiple host species (Cleaveland et al., 2001; Gilbert et al., 2012; Gilbert & Webb, 2007; Malpica et al., 2006; Novotny et al., 2002; Pearse & Hipp, 2009; Weiblen et al., 2006), these essential evolutionary tools in species conservation efforts are also broadly applicable. For domesticated mammals, Farrell and Davies (2019) demonstrated that evolutionary distance from an infected host to another mammal host species is a strong predictor of multi‐host disease‐induced mortality. Similarly, Gilbert et al. (2015) reported that the relative amount of damage done by a natural enemy on plant species declines predictably with increasing evolutionary distance from highly susceptible hosts. Our study affirms that the use of host evolutionary relationships presents a unifying theoretical approach to predicting disease outcomes across multiple host‐pest combinations.

### 4.3 Epidemiological implications of host evolutionary relationships

In addition to determining which species are prone to pest‐induced mortality, host evolutionary relationships can be used to understand complex epidemiological outcomes and help prioritize surveillance activities in vulnerable, naïve communities. For FD‐ISHB, the stronger phylogenetic effects with increasingly severe host impacts correspond to potential epidemiological outcomes. These outcomes are likely consistent with stages of invasion in which non‐competent hosts may foster beetle arrival to a new area, competent hosts facilitate beetle‐fungal establishment and pest‐pathogen persistence, and killed‐competent hosts correspond to pest‐pathogen spread and ecosystems impact. Because FD‐ISHB non‐competent hosts exhibit a phylogenetic signal, beetle arrival most likely corresponds to a broad suite of polygenic traits that attract beetles to trees, but other trait aggregates that confer induced defence can prevent beetle establishment. This phenomenon has been demonstrated for two conspecific cultivars of tea (*Camellia sinensis*) with different susceptibilities to *Euwallacea perbrevis* in Sri Lanka (Karunaratne et al., 2009). Both cultivars are equally attractive to beetle attack, but while beetles established galleries in the susceptible cultivar, they abandoned partly bored galleries the resistant cultivar, suggesting beetle attack induced plant defences in the resistant cultivar. In systems with such ecological stepping stones of hosts of different susceptibility, a larger pool of closely related susceptible species in a local plant community increases a beetle's chance of encountering a competent host individual; non‐competent hosts that do not kill the beetle may therefore facilitate establishment in a new location through contact with individuals representing closely related competent host species.

The even more phylogenetically constrained competent hosts that survive attack represent a low virulence interaction that promotes pest‐pathogen persistence in reservoir hosts. The most severely affected competent hosts represent a high‐virulence interaction, show the most striking phylogenetic effect and largely correspond to pest‐pathogen spread. Young adult *Euwallacea* females emerging from native galleries prefer to produce and remain in their natal galleries on the same individual tree (Calnaido & Thirugnanasuntharau, 1966; Lynch et al., 2019). Population propagules thus amplify over time until the dying host can no longer support beetle reproduction and beetles escape the tree in a mass dispersal event, aiding in the epidemic spread of the pest–pathogen complex. Thus, our study demonstrates that understanding epidemiological outcomes based on the phylogenetic structure of the nested outcomes of multi‐host pest interactions can help determine which species contribute to different stages of an invasion process.

To optimize the use of limited resources, an understanding of host evolutionary relationships can be utilized to stratify survey efforts and focus on areas with different combinations of species representing groups that appear to be most important in the arrival, establishment and spread of the pest–pathogen complex. For example, surveys of wildland forests in South Africa could prioritize locations comprising some combination of species in the Fabaceae, Salicaceae and Sapindaceae, which are common in South Africa (http://pza.sanbi.org/vegetation) and consist of many host species important to all stages of an invasion. Common species in families with many hosts important to beetle arrival (e.g., Podocarpaceae, Proteaceae, Myrtaceae) or establishment (e.g., Myrtaceae, Arecaceae) could also be prioritized. Another way to prioritize survey efforts could be to target species belonging to the genus *Dombeya* (Malvaceae), given that many naturally occur in South Africa but not California, and *D*. *cacuminum* is a competent host. Targeting species belonging to Annonaceae or Strelitziaceae would be of low priority since these families do not contain host species and are found outside the more susceptible Rosid clade.

### 4.4 Caveats

One limitation to our analysis is that our information on which hosts the *Fusarium* pathogens can grow is not independent of beetle attack. Experimental inoculations of the fungi on confirmed nonhost tree species (no symptoms of beetle attack) would indicate whether the *Fusarium* host range is truly constrained phylogenetically. However, the relationship between the beetles and their fungi is tightly coupled. The *Fusarium* species belong to the monophyletic Ambrosia Fusarium Clade (AFC; Kasson et al., 2013) and the ~22 Myr old mutualism between AFC members and beetles in the genus *Euwallacea* represents 1 of 11 known evolutionary origins of fungiculture by ambrosia beetles (O’Donnell et al., 2015). These closely related wood‐inhabiting *Fusarium* species are transmitted in mycangia and cultivated by females in galleries as a source of nutrition for the beetle (Kasson et al., 2013; O’Donnell et al., 2015). Key survival structures of the *Fusarium* species that aid in their dispersal have not been observed on *Fusarium*‐colonized non‐competent hosts, which suggests that their chance of spread without their beetle vector is very low. Therefore, fungal colonization on artificially inoculated plant species outside the phylogenetic constraints of beetle‐attacked species may not be as important as the beetle‐fungal‐host interactions combined.

The strength of the phylogenetic signal seen between different host types provides a working hypothesis as to which species we expect to be new hosts prone to different levels of *Fusarium*‐ISHB attack in South Africa. Our California data set is based on 8 years of comprehensive and ongoing surveys throughout the infested region, representing the most complete host list available. However, the host list includes additional species in new families based on preliminary surveys in South Africa, which do not occur in California (Calflora, 2020); https://www.fabinet.up.ac.za/pshb). New species include one new competent host in a new Malvid family within the Rosid clade (Combretaceae: *Combretum kraussii*), and three non‐competent hosts representing two new families outside the Rosids (Primulaceae: *Rapanea melanophloeos*; Stilbaceae: *Halleria lucida* and *Nuxia floribunda*). Other new families with non‐competent host genera that do not occur in California include Primulaceae (*Rapanea*), Boraginaceae (*Cordia*) and Celastraceae (*Gymnosporia*); all but the latter occur outside the Rosid clade. Interestingly, Aoki et al. (2018) observed attacks by *Euwallacea validus* on tree species in the eastern USA that occur within the same highly phylogenetically constrained Fabid and Malvid groups as the ISHB beetles. Additionally, all three beetle species (*E*.* validus*, *E*.* fornicatus*, *E*.* kuroshio*) share at least seven orders containing competent hosts. Together with all seven new competent host species clumping within the Rosids, and the remaining additional six competent and 19 non‐competent host species clustering within existing groups, we can conclude that the overall phylogenetic patterns hold for the growing host list and potentially for host ranges of other *Euwallacea*‐AFC members.

Phylogenetic models based on evolutionary distances between hosts of generalist pests can be used to evaluate which host species are potentially most vulnerable to pest impacts and most important to their establishment and spread. Certainly, other essential factors that drive host‐pest interactions influence host outcomes. Changes in environmental conditions, pathogen virulence or the host microbiome can amplify or inhibit host susceptibility or damage. In particular, the phylogenetic structure and host abundance of local communities strongly influence the severity of impact on focal hosts (Parker et al., 2015). Although phylogenetic signal in host range cannot fully explain overall epidemic patterns, it can be used as a first approximation to understanding complex novel pest invasions, serving as a powerful tool to assess risk and guide response priorities.

## CONFLICT OF INTEREST

The authors confirm that they do not have any conflicts of interest to declare.

## Supporting information

Fig S1

Fig S2

Fig S3

Table S1

Data S1

## Data Availability

The authors confirm that the data supporting the findings of this study are available within the article and its Supporting information.
