# Supplementary figures and images for: Host evolutionary relationships explain tree mortality caused by a generalist pest–pathogen complex
Source: Evol Appl. 2021 Jan 5;14(4):1083–94. doi: 10.1111/eva.13182 (PMC8061262; doi:10.1111/eva.13182)

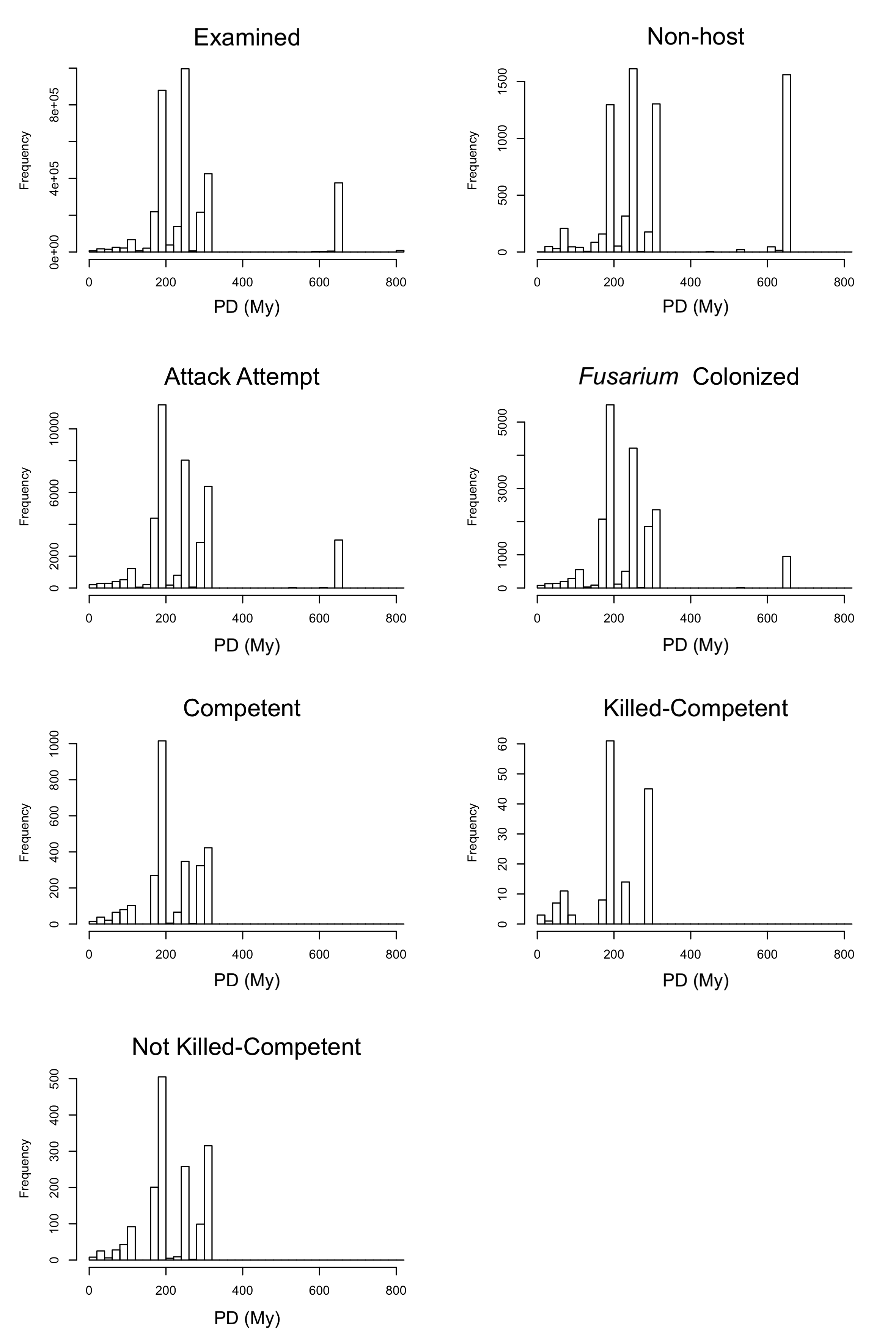

Supplement: Supplementary file 1 — Fig S1 [file EVA-14-1083-s003.png]

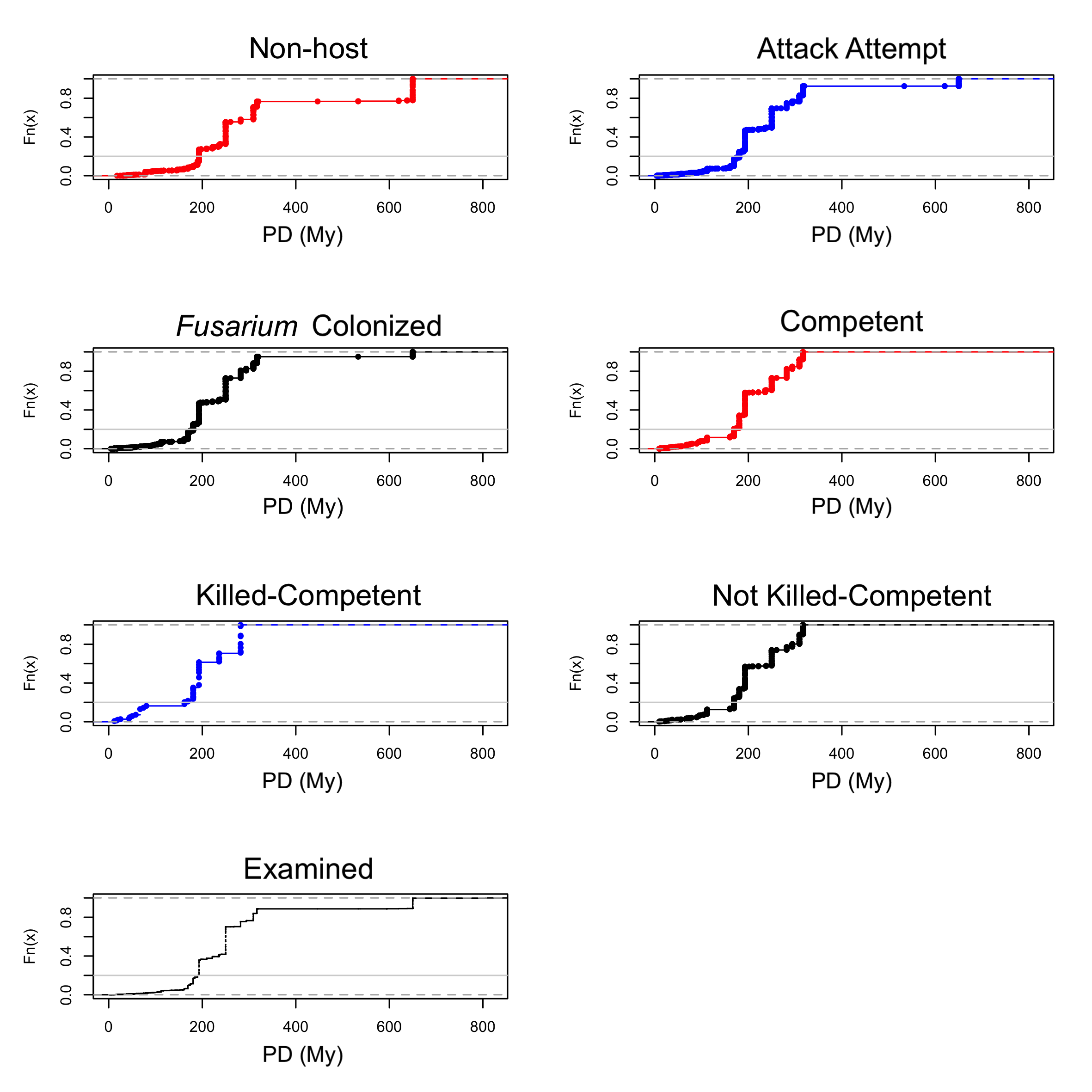

Supplement: Supplementary file 2 — Fig S2 [file EVA-14-1083-s001.png]

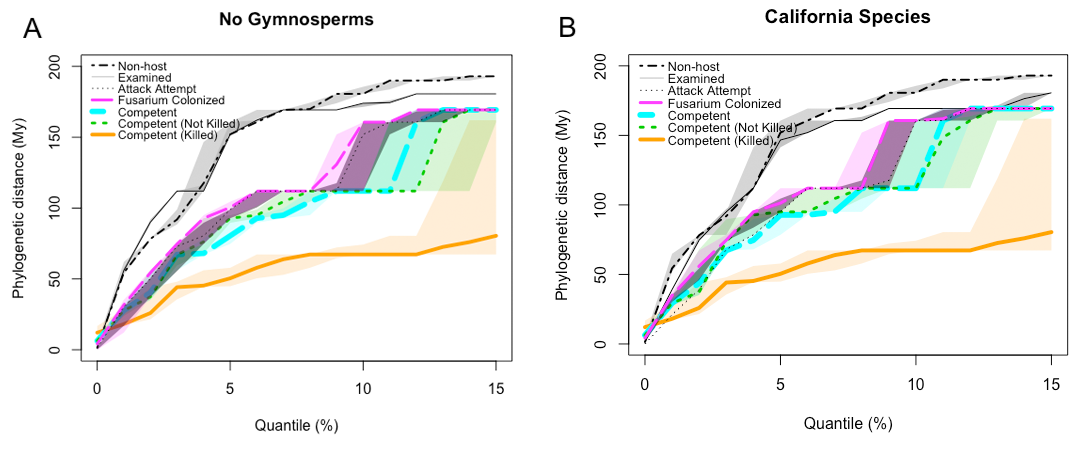

Supplement: Supplementary file 3 — Fig S3 [file EVA-14-1083-s005.png]
