# Supplementary material for: Host evolutionary relationships explain tree mortality caused by a generalist pest–pathogen complex
Source: Evol Appl. 2021 Jan 5;14(4):1083–94. doi: 10.1111/eva.13182 (PMC8061262; doi:10.1111/eva.13182)
Supplement: Supplementary file 4 — Table S1 [file EVA-14-1083-s004.docx]

Supplementary Table I. Native range and infested distribution (California—CA or South Africa—ZA) of tree and shrub ISHB host species organized phylogenetically from the most to least ancestral lineages. Non-competent hosts are either attacked by the beetle species alone (A) or attacked by beetles and colonized by *Fusarium* spp. (F). Competent hosts support beetle-pathogen reproduction and are killed (K) or not killed (NK) when attacked.

| Order | Family | Species | Non-Competent | Competent | Native Range | Infested Distribution |
| --- | --- | --- | --- | --- | --- | --- |
| Pinales | Cupressaceae | *Juniperus chinensis* | F |  |  | CA |
| Pinales | Cupressaceae | *Juniperus virginiana* | A |  |  | CA |
| Pinales | Cupressaceae | *Metasequoia glyptostroboides* | F |  |  | CA |
| Pinales | Cupressaceae | *Taxodium distichum* | F |  |  | CA & ZA |
| Pinales | Podocarpaceae | *Afrocarpus falcatus* | F |  | ZA | ZA |
| Pinales | Podocarpaceae | *Afrocarpus gracilior* | A |  |  | CA |
| Pinales | Podocarpaceae | *Podocarpus henkelii* | F |  | ZA | CA & ZA |
| Pinales | Pinaceae | *Cedrus atlantica* | A |  |  | CA |
| Pinales | Pinaceae | *Keteleeria evelyniana* | A |  |  | CA |
| Pinales | Pinaceae | *Pinus densiflora* | A |  |  | CA |
| Pinales | Pinaceae | *Pinus douglasiana* | A |  |  | CA |
| Laurales | Lauraceae | *Beilschmiedia miersii* | A |  |  | CA |
| Laurales | Lauraceae | *Cinnamomum camphora* | F |  |  | CA & ZA |
| Laurales | Lauraceae | *Cinnamomum glanduliferum* | A |  |  | CA |
| Laurales | Lauraceae | *Machilus thunbergii* | F |  |  | CA |
| Laurales | Lauraceae | *Nothaphoebe sp.* | A |  |  | CA |
| Laurales | Lauraceae | *Persea americana* |  | NK |  | CA & ZA |
| Laurales | Lauraceae | *Umbellularia californica* | F |  | CA | CA |
| Laurales | Monimiaceae | *Peumus boldus* | A |  |  | CA |
| Magnoliales | Magnoliaceae | *Liriodendron tulipifera* | F |  |  | CA |
| Magnoliales | Magnoliaceae | *Magnolia delavayi* | A |  |  | CA |
| Magnoliales | Magnoliaceae | *Magnolia doltsopa* | A |  |  | CA |
| Supplementary Table I. Continued. | | | | | | |
| Order | Family | Species | Non-Competent | Competent | Native Range | Infested Distribution |
| Magnoliales | Magnoliaceae | *Magnolia grandiflora* |  | NK |  | CA & ZA |
| Magnoliales | Magnoliaceae | *Magnolia guatemalensis* | A |  |  | CA |
| Magnoliales | Magnoliaceae | *Magnolia soulangeana* | A |  |  | CA |
| Magnoliales | Magnoliaceae | *Magnolia* sp. | A |  |  | CA |
| Magnoliales | Magnoliaceae | *Magnolia veitchii* | F |  |  | CA |
| Magnoliales | Magnoliaceae | *Magnolia virginiana* |  | NK |  | CA |
| Poales | Poaceae | *Bambusa oldhamii* | A |  |  | CA |
| Poales | Poaceae | *Bambusa* sp. | A |  |  | CA |
| Arecales | Arecaceae | *Archontophoenix cunninghamiana* |  | NK |  | CA |
| Arecales | Arecaceae | *Butia capitata* | A |  |  | CA |
| Arecales | Arecaceae | *Chamaedorea elegans* | F |  |  | CA |
| Arecales | Arecaceae | *Howea forsteriana* |  | NK |  | CA |
| Arecales | Arecaceae | *Livistona chinensis* | F |  |  | CA |
| Arecales | Arecaceae | *Ptychosperma elegans* |  | NK |  | CA |
| Arecales | Arecaceae | *Washingtonia filifera* | F |  | CA | CA |
| Arecales | Arecaceae | *Washingtonia robusta* | A |  |  | CA |
| Asparagales | Asparagaceae | *Dracaena draco* | A |  |  | CA |
| Ranunculales | Menispermaceae | *Cocculus laurifolius* |  | NK |  | CA |
| Ranunculales | Menispermaceae | *Cocculus orbiculatus* | F |  |  | CA |
| Ranunculales | Papaveraceae | *Bocconia arborea* | A |  |  | CA |
| Proteales | Proteaceae | *Banksia saxicola* | F |  |  | CA |
| Proteales | Proteaceae | *Macadamia integrifolia* | F |  |  | CA & ZA |
| Proteales | Proteaceae | *Protea mundii* | F |  | ZA | ZA |
| Proteales | Platanaceae | *Platanus acerifolia* |  | K |  | CA & ZA |
| Supplementary Table I. Continued. | | | | | | |
| Order | Family | Species | Non-Competent | Competent | Native Range | Infested Distribution |
| Proteales | Platanaceae | *Platanus hispanica* |  | K |  | CA |
| Proteales | Platanaceae | *Platanus mexicana* |  | NK |  | CA |
| Proteales | Platanaceae | *Platanus occidentalis* | F |  |  | CA & ZA |
| Proteales | Platanaceae | *Platanus racemosa* |  | K | CA | CA & ZA |
| Proteales | Platanaceae | *Platanus wrightii* | F |  |  | CA |
| Caryophyllales | Tamaricaceae | *Tamarix ramosissima* |  | NK |  | CA |
| Cornales | Cornaceae | *Alangium sp.* | A |  |  | CA |
| Cornales | Cornaceae | *Camptotheca acuminata* | F |  |  | CA |
| Cornales | Cornaceae | *Cornus controversa* | F |  |  | CA |
| Ericales | Pentaphylacaceae | *Cleyera japonica* | F |  |  | CA |
| Ericales | Ebenaceae | *Diospyros dichrophylla* | F |  | ZA | ZA |
| Ericales | Ebenaceae | *Diospyros kaki* | A |  |  | CA |
| Ericales | Ebenaceae | *Diospyros lycioides* | F |  | ZA | ZA |
| Ericales | Primulaceae | *Rapanea melanophloeos* | F |  | ZA | ZA |
| Ericales | Theaceae | *Camellia japonica* | F |  |  | CA & ZA |
| Ericales | Theaceae | *Camellia reticulata* | F |  |  | CA |
| Ericales | Theaceae | *Camellia semiserrata* |  | NK |  | CA |
| Ericales | Ericaceae | *Arbutus unedo* | A |  |  | CA |
| Aquifoliales | Aquifoliaceae | *Ilex cornuta* |  | NK |  | CA |
| Aquifoliales | Aquifoliaceae | *Ilex latifolia* | F |  |  | CA |
| Asterales | Asteraceae | *Baccharis pilularis* |  | NK |  | CA |
| Asterales | Asteraceae | *Baccharis salicina* |  | NK | CA | CA |
| Asterales | Asteraceae | *Verbesina gigantea* | A |  |  | CA |
| Dipsacales | Adoxaceae | *Viburnum sinensis* | F |  |  | ZA |
| Supplementary Table I. Continued. | | | | | | |
| Order | Family | Species | Non-Competent | Competent | Native Range | Infested Distribution |
| Apiales | Pittosporaceae | *Hymenosporum flavum* | F |  |  | CA |
| Apiales | Pittosporaceae | *Pittosporum undulatum* | F |  |  | CA |
| Apiales | Araliaceae | *Cussonia spicata* | F |  | ZA | CA & ZA |
| Apiales | Araliaceae | *Fatsia japonica* | F |  |  | CA |
| Boraginales | Boraginaceae | *Cordia caffra* | F |  | ZA | ZA |
| Boraginales | Boraginaceae | *Wigandia urens* | A |  |  | CA |
| Lamiales | Oleaceae | *Chionanthus retusus* | F |  |  | CA |
| Lamiales | Oleaceae | *Fraxinus excelsior* | F |  |  | CA & ZA |
| Lamiales | Oleaceae | *Fraxinus uhdei* | F |  |  | CA |
| Lamiales | Oleaceae | *Fraxinus velutina* | A |  | CA | CA |
| Lamiales | Oleaceae | *Olea europaea* | F |  |  | CA & ZA |
| Lamiales | Oleaceae | *Olea sp.* | F |  | ZA | ZA |
| Lamiales | Oleaceae | *Osmanthus fragrans* | A |  |  | CA |
| Lamiales | Scrophulariaceae | *Buddleja saligna* | F |  | ZA | ZA |
| Lamiales | Scrophulariaceae | *Myoporum laetum* | F |  |  | CA |
| Lamiales | Stilbaceae | *Halleria lucida* | F |  | ZA | ZA |
| Lamiales | Stilbaceae | *Nuxia floribunda* | F |  | ZA | CA & ZA |
| Lamiales | Bignoniaceae | *Catalpa speciosa* | F |  |  | CA |
| Lamiales | Bignoniaceae | *Handroanthus impetiginosus* | A |  |  | CA |
| Lamiales | Bignoniaceae | *Jacaranda mimosifolia* |  | NK |  | CA & ZA |
| Lamiales | Bignoniaceae | *Markhamia lutea* | A |  |  | CA |
| Lamiales | Bignoniaceae | *Spathodea campanulata* |  | NK |  | CA |
| Lamiales | Verbenaceae | *Aloysia sp.* | F |  |  | CA |
| Gentianales | Apocynaceae | *Cascabela thevetioides* | A |  |  | CA |
| Supplementary Table I. Continued. | | | | | | |
| Order | Family | Species | Non-Competent | Competent | Native Range | Infested Distribution |
| Gentianales | Apocynaceae | *Plumeria rubra* | F |  |  | CA & ZA |
| Saxifragales | Altingiaceae | *Liquidambar formosana* | F |  |  | CA |
| Saxifragales | Altingiaceae | *Liquidambar styraciflua* |  | K |  | CA & ZA |
| Vitales | Vitaceae | *Vitis vinifera* | F |  |  | CA & ZA |
| Celastrales | Celastraceae | *Gymnosporia buxifolia* | F |  | ZA | ZA |
| Oxalidales | Cunoniaceae | *Cunonia capensis* | A |  |  | CA & ZA |
| Oxalidales | Elaeocarpaceae | *Crinodendron patagua* | A |  |  | CA |
| Malpighiales | Phyllanthaceae | *Bischofia javanica* | F |  |  | CA |
| Malpighiales | Salicaceae | *Dovyalis caffra* | F |  |  | CA & ZA |
| Malpighiales | Salicaceae | *Populus fremontii* |  | K | CA | CA |
| Malpighiales | Salicaceae | *Populus nigra* |  | K |  | CA & ZA |
| Malpighiales | Salicaceae | *Populus tremuloides* |  | NK | CA | CA |
| Malpighiales | Salicaceae | *Populus trichocarpa* |  | K | CA | CA |
| Malpighiales | Salicaceae | *Salix alba* |  | NK |  | ZA |
| Malpighiales | Salicaceae | *Salix babylonica* |  | NK |  | CA |
| Malpighiales | Salicaceae | *Salix exigua* | F |  | CA | CA |
| Malpighiales | Salicaceae | *Salix gooddingii* |  | K | CA | CA |
| Malpighiales | Salicaceae | *Salix laevigata* |  | K | CA | CA |
| Malpighiales | Salicaceae | *Salix lasiolepis* |  | K | CA | CA |
| Malpighiales | Salicaceae | *Salix mucronata* |  | NK | ZA | ZA |
| Malpighiales | Salicaceae | *Xylosma congesta* |  | NK |  | CA |
| Malpighiales | Euphorbiaceae | *Jatropha cinerea* | F |  |  | CA |
| Malpighiales | Euphorbiaceae | *Manihot esculenta* | A |  |  | CA |
| Malpighiales | Euphorbiaceae | *Ricinus communis* |  | K |  | CA & ZA |
| Supplementary Table I. Continued. | | | | | | |
| Order | Family | Species | Non-Competent | Competent | Native Range | Infested Distribution |
| Malpighiales | Euphorbiaceae | *Triadica sebifera* | A |  |  | CA |
| Malpighiales | Euphorbiaceae | *Vernicia fordii* | A |  |  | CA |
| Rosales | Rosaceae | *Chaenomeles sinensis* | A |  |  | CA |
| Rosales | Rosaceae | *Eriobotrya japonica* | F |  |  | CA & ZA |
| Rosales | Rosaceae | *Malus floribunda* | A |  |  | CA |
| Rosales | Rosaceae | *Prunus africana* | F |  | ZA | ZA |
| Rosales | Rosaceae | *Prunus caroliniana* | A |  |  | CA |
| Rosales | Rosaceae | *Prunus cerasoides* | A |  |  | CA |
| Rosales | Rosaceae | *Prunus mume* | F |  |  | CA |
| Rosales | Rosaceae | *Prunus nigra* | F |  |  | ZA |
| Rosales | Rosaceae | *Prunus persica* | F |  |  | CA & ZA |
| Rosales | Rosaceae | *Prunus serrulata* | F |  |  | CA |
| Rosales | Rosaceae | *Pyrus calleryana* | F |  |  | CA |
| Rosales | Rosaceae | *Pyrus kawakamii* | F |  |  | CA |
| Rosales | Ulmaceae | *Ulmus alata* | F |  |  | CA |
| Rosales | Ulmaceae | *Ulmus americana* | F |  |  | CA |
| Rosales | Ulmaceae | *Ulmus minor* | F |  |  | CA & ZA |
| Rosales | Ulmaceae | *Ulmus parvifolia* | F |  |  | CA & ZA |
| Rosales | Ulmaceae | *Zelkova serrata* | F |  |  | CA |
| Rosales | Rhamnaceae | *Frangula californica* | A |  | CA | CA |
| Rosales | Rhamnaceae | *Ziziphus jujuba* | F |  |  | CA |
| Rosales | Moraceae | *Broussonetia papyrifera* | A |  |  | CA |
| Rosales | Moraceae | *Ficus altissima* |  | NK |  | CA |
| Rosales | Moraceae | *Ficus benjamina* | A |  |  | CA |
| Supplementary Table I. Continued. | | | | | | |
| Order | Family | Species | Non-Competent | Competent | Native Range | Infested Distribution |
| Rosales | Moraceae | *Ficus carica* |  | NK |  | CA & ZA |
| Rosales | Moraceae | *Ficus macrophylla* | F |  |  | CA |
| Rosales | Moraceae | *Ficus maxima* | A |  |  | CA |
| Rosales | Moraceae | *Ficus natalensis* | F |  | ZA | ZA |
| Rosales | Moraceae | *Ficus platypoda* | F |  |  | CA |
| Rosales | Moraceae | *Morus alba* | F |  |  | CA & ZA |
| Rosales | Urticaceae | *Pipturus argenteus* | F |  |  | CA |
| Fagales | Fagaceae | *Fagus crenata* |  | NK |  | CA |
| Fagales | Fagaceae | *Fagus sylvatica* | F |  |  | CA |
| Fagales | Fagaceae | *Quercus acutidens* | A |  | CA | CA |
| Fagales | Fagaceae | *Quercus acutissima* | A |  |  | CA |
| Fagales | Fagaceae | *Quercus agrifolia* |  | NK | CA | CA |
| Fagales | Fagaceae | *Quercus alba* | A |  |  | CA |
| Fagales | Fagaceae | *Quercus buckleyi* | A |  |  | CA |
| Fagales | Fagaceae | *Quercus chrysolepis* |  | NK | CA | CA |
| Fagales | Fagaceae | *Quercus coccinea* | A |  |  | CA |
| Fagales | Fagaceae | *Quercus engelmannii* |  | NK | CA | CA |
| Fagales | Fagaceae | *Quercus ilex* | F |  |  | CA |
| Fagales | Fagaceae | *Quercus lobata* |  | K | CA | CA |
| Fagales | Fagaceae | *Quercus macrocarpa* |  | NK |  | CA |
| Fagales | Fagaceae | *Quercus mexicana* | F |  |  | CA |
| Fagales | Fagaceae | *Quercus palustris* | F |  |  | CA & ZA |
| Fagales | Fagaceae | *Quercus polymorpha* | A |  |  | CA |
| Fagales | Fagaceae | *Quercus robur* |  | K |  | CA & ZA |
| Supplementary Table I. Continued. | | | | | | |
| Order | Family | Species | Non-Competent | Competent | Native Range | Infested Distribution |
| Fagales | Fagaceae | *Quercus rubra* | A |  |  | CA |
| Fagales | Fagaceae | *Quercus rugosa* | A |  |  | CA |
| Fagales | Fagaceae | *Quercus shumardii* | F |  |  | CA |
| Fagales | Fagaceae | *Quercus suber* |  | NK |  | CA |
| Fagales | Fagaceae | *Quercus virginiana* | F |  |  | CA |
| Fagales | Fagaceae | *Quercus wislizeni* | A |  | CA | CA |
| Fagales | Juglandaceae | *Carya illinoinensis* | F |  |  | CA & ZA |
| Fagales | Juglandaceae | *Juglans mandshurica* | A |  |  | CA |
| Fagales | Juglandaceae | *Juglans nigra* | A |  |  | CA |
| Fagales | Juglandaceae | *Pterocarya sp.* | A |  |  | CA |
| Fagales | Juglandaceae | *Pterocarya stenoptera* |  | NK |  | CA |
| Fagales | Betulaceae | *Alnus incana* | A |  | CA | CA |
| Fagales | Betulaceae | *Alnus rhombifolia* |  | NK | CA | CA |
| Fagales | Betulaceae | *Betula pendula* | F |  | ZA | CA & ZA |
| Fagales | Betulaceae | *Corylus colurna* | F |  |  | CA |
| Fagales | Casuarinaceae | *Casuarina cunninghamiana* | F |  |  | CA |
| Fagales | Casuarinaceae | *Casuarina equisetifolia* |  | NK |  | CA |
| Fabales | Fabaceae | *Acacia aneura* | F |  |  | CA |
| Fabales | Fabaceae | *Acacia baileyana* | F |  |  | CA |
| Fabales | Fabaceae | *Acacia floribunda* | A |  |  | CA |
| Fabales | Fabaceae | *Acacia mearnsii* |  | NK |  | ZA |
| Fabales | Fabaceae | *Acacia melanoxylon* |  | NK |  | CA & ZA |
| Fabales | Fabaceae | *Acacia saligna* | A |  |  | CA |
| Fabales | Fabaceae | *Acacia sp.* |  | NK |  | CA |
| Supplementary Table I. Continued. | | | | | | |
| Order | Family | Species | Non-Competent | Competent | Native Range | Infested Distribution |
| Fabales | Fabaceae | *Acacia stenophylla* | A |  |  | CA |
| Fabales | Fabaceae | *Acacia victoriae* | A |  |  | CA |
| Fabales | Fabaceae | *Albizia gummifera* | A |  |  | CA |
| Fabales | Fabaceae | *Albizia julibrissin* |  | NK |  | CA |
| Fabales | Fabaceae | *Albizia kalkora* | A |  |  | CA |
| Fabales | Fabaceae | *Bauhinia blakeana* | F |  |  | CA |
| Fabales | Fabaceae | *Bauhinia galpinii* | F |  | ZA | CA & ZA |
| Fabales | Fabaceae | *Bauhinia petersiana* | A |  |  | CA & ZA |
| Fabales | Fabaceae | *Bauhinia purpurea* | F |  |  | ZA |
| Fabales | Fabaceae | *Bauhinia variegata* |  | NK |  | CA |
| Fabales | Fabaceae | *Caesalpinia cacalaco* | A |  |  | CA |
| Fabales | Fabaceae | *Calpurnia aurea* | F |  | ZA | CA & ZA |
| Fabales | Fabaceae | *Cassia abbreviata* | A |  | ZA | CA & ZA |
| Fabales | Fabaceae | *Cassia brewsteri* | F |  |  | CA |
| Fabales | Fabaceae | *Cassia fistula* | A |  |  | CA |
| Fabales | Fabaceae | *Cassia leptophylla* | F |  |  | CA |
| Fabales | Fabaceae | *Castanospermum australe* |  | NK |  | CA |
| Fabales | Fabaceae | *Ceratonia siliqua* | F |  |  | CA |
| Fabales | Fabaceae | *Cercidium floridum subsp. floridum* |  | NK | CA | CA |
| Fabales | Fabaceae | *Cercidium microphyllum* | A |  | CA | CA |
| Fabales | Fabaceae | *Cercidium sonorae* |  | NK |  | CA |
| Fabales | Fabaceae | *Cercidium* sp. 1 | A |  |  | CA |
| Fabales | Fabaceae | *Cladrastis sinensis* | A |  |  | CA |
| Fabales | Fabaceae | *Dalbergia sissoo* | F |  |  | CA |
| Supplementary Table I. Continued. | | | | | | |
| Order | Family | Species | Non-Competent | Competent | Native Range | Infested Distribution |
| Fabales | Fabaceae | *Erythrina abyssinica* | A |  |  | CA |
| Fabales | Fabaceae | *Erythrina caffra* |  | NK | ZA | CA & ZA |
| Fabales | Fabaceae | *Erythrina coralloides* |  | NK |  | CA |
| Fabales | Fabaceae | *Erythrina crista-galli* | F |  |  | CA |
| Fabales | Fabaceae | *Erythrina falcata* |  | NK |  | CA |
| Fabales | Fabaceae | *Erythrina folkersii* | F |  |  | CA |
| Fabales | Fabaceae | *Erythrina humeana* | F |  |  | CA & ZA |
| Fabales | Fabaceae | *Erythrina livingstoniana* | F |  |  | ZA |
| Fabales | Fabaceae | *Erythrina lysistemon* | F |  | ZA | CA & ZA |
| Fabales | Fabaceae | *Erythrina sykesii* | A |  |  | CA |
| Fabales | Fabaceae | *Gleditsia triacanthos* |  | NK |  | CA & ZA |
| Fabales | Fabaceae | *Inga edulis* | F |  |  | CA |
| Fabales | Fabaceae | *Inga feuilleei* | A |  |  | CA |
| Fabales | Fabaceae | *Lysiphyllum carronii* | F |  |  | CA |
| Fabales | Fabaceae | *Olneya tesota* | A |  | CA | CA |
| Fabales | Fabaceae | *Parkinsonia aculeata* |  | K |  | CA |
| Fabales | Fabaceae | *Pithecellobium sp.* | A |  |  | CA |
| Fabales | Fabaceae | *Podalyria calyptrata* |  | NK | ZA | ZA |
| Fabales | Fabaceae | *Prosopis articulata* |  | NK |  | CA |
| Fabales | Fabaceae | *Prosopis chilensis* | F |  |  | CA |
| Fabales | Fabaceae | *Prosopis glandulosa* | F |  |  | CA |
| Fabales | Fabaceae | *Prosopis velutina* | F |  |  | CA |
| Fabales | Fabaceae | *Psoralea pinnata* |  | NK | ZA | ZA |
| Fabales | Fabaceae | *Schotia brachypetala* | F |  | ZA | CA & ZA |
| Supplementary Table I. Continued. | | | | | | |
| Order | Family | Species | Non-Competent | Competent | Native Range | Infested Distribution |
| Fabales | Fabaceae | *Senegalia caffra* | A |  |  | CA & ZA |
| Fabales | Fabaceae | *Senegalia galpinii* | F |  | ZA | ZA |
| Fabales | Fabaceae | *Senegalia visco* | F |  |  | CA |
| Fabales | Fabaceae | *Senna racemosa* | F |  |  | CA |
| Fabales | Fabaceae | *Senna spectabilis* | A |  |  | CA |
| Fabales | Fabaceae | *Senna splendida* | F |  |  | CA |
| Fabales | Fabaceae | *Styphnolobium japonicum* | A |  |  | CA |
| Fabales | Fabaceae | *Tipuana tipu* | A |  |  | CA |
| Fabales | Fabaceae | *Vachellia caven* | A |  |  | CA |
| Fabales | Fabaceae | *Vachellia cochliacantha* | A |  |  | CA |
| Fabales | Fabaceae | *Vachellia etbaica* | A |  |  | CA |
| Fabales | Fabaceae | *Vachellia farnesiana* | F |  |  | CA |
| Fabales | Fabaceae | *Vachellia karroo* | F |  | ZA | ZA |
| Fabales | Fabaceae | *Vachellia sieberiana* | F |  | ZA | ZA |
| Fabales | Fabaceae | *Virgilia divaricata* | F |  | ZA | ZA |
| Fabales | Fabaceae | *Virgilia oroboides* |  | NK | ZA | ZA |
| Fabales | Fabaceae | *Wisteria floribunda* |  | NK |  | CA |
| Fabales | Fabaceae | *Wisteria sinensis* | F |  |  | CA |
| Fabales | Fabaceae | *Zenia insignis* | A |  |  | CA |
| Geraniales | Melianthaceae | *Melianthus major* | F |  | ZA | CA & ZA |
| Myrtales | Combretaceae | *Combretum erythrophyllum* | F |  | ZA | ZA |
| Myrtales | Combretaceae | *Combretum kraussii* |  | NK | ZA | ZA |
| Myrtales | Onagraceae | *Hauya elegans* | A |  |  | CA |
| Myrtales | Myrtaceae | *Callistemon salignus* | A |  |  | CA |
| Supplementary Table I. Continued. | | | | | | |
| Order | Family | Species | Non-Competent | Competent | Native Range | Infested Distribution |
| Myrtales | Myrtaceae | *Callistemon viminalis* | A |  |  | CA |
| Myrtales | Myrtaceae | *Corymbia ficifolia* |  | NK |  | CA |
| Myrtales | Myrtaceae | *Eucalyptus camaldulensis* | F |  |  | CA & ZA |
| Myrtales | Myrtaceae | *Eucalyptus cinerea* | A |  |  | CA |
| Myrtales | Myrtaceae | *Eucalyptus froggattii* | A |  |  | CA |
| Myrtales | Myrtaceae | *Eucalyptus kitsoniana* | A |  |  | CA |
| Myrtales | Myrtaceae | *Eucalyptus perriniana* | A |  |  | CA |
| Myrtales | Myrtaceae | *Eucalyptus polyanthemos* | F |  |  | CA |
| Myrtales | Myrtaceae | *Eucalyptus torquata* | F |  |  | CA |
| Myrtales | Myrtaceae | *Psidium guajava* | F |  |  | CA & ZA |
| Sapindales | Anacardiaceae | *Harpephyllum caffrum* | F |  | ZA | CA & ZA |
| Sapindales | Anacardiaceae | *Pistacia chinensis* | A |  |  | CA |
| Sapindales | Anacardiaceae | *Schinus molle* | F |  |  | CA & ZA |
| Sapindales | Anacardiaceae | *Schinus polygama* | F |  |  | ZA |
| Sapindales | Anacardiaceae | *Schinus terebinthifolia* | F |  |  | CA |
| Sapindales | Burseraceae | *Bursera hindsiana* | A |  |  | CA |
| Sapindales | Sapindaceae | *Acer buergerianum* |  | K |  | CA & ZA |
| Sapindales | Sapindaceae | *Acer caudatifolium* | A |  |  | CA |
| Sapindales | Sapindaceae | *Acer davidii* | A |  |  | CA |
| Sapindales | Sapindaceae | *Acer freemanii* | A |  |  | CA |
| Sapindales | Sapindaceae | *Acer macrophyllum* |  | K | CA | CA |
| Sapindales | Sapindaceae | *Acer negundo* |  | K | CA | CA & ZA |
| Sapindales | Sapindaceae | *Acer palmatum* |  | K |  | CA & ZA |
| Sapindales | Sapindaceae | *Acer paxii* |  | NK |  | CA |
| Supplementary Table I. Continued. | | | | | | |
| Order | Family | Species | Non-Competent | Competent | Native Range | Infested Distribution |
| Sapindales | Sapindaceae | *Acer pectinatum* | F |  |  | CA |
| Sapindales | Sapindaceae | *Aesculus californica* |  | NK | CA | CA |
| Sapindales | Sapindaceae | *Alectryon excelsus* |  | NK |  | CA |
| Sapindales | Sapindaceae | *Cupaniopsis anacardioides* |  | NK |  | CA |
| Sapindales | Sapindaceae | *Harpullia arborea* | F |  |  | CA |
| Sapindales | Sapindaceae | *Harpullia pendula* |  | NK |  | CA |
| Sapindales | Sapindaceae | *Koelreuteria bipinnata* |  | NK |  | CA |
| Sapindales | Sapindaceae | *Koelreuteria elegans* | F |  |  | CA |
| Sapindales | Sapindaceae | *Koelreuteria paniculata* | F |  |  | CA |
| Sapindales | Sapindaceae | *Ungnadia speciosa* | F |  |  | CA |
| Sapindales | Meliaceae | *Aglaia odorata* | A |  |  | CA |
| Sapindales | Meliaceae | *Ekebergia capensis* | F |  | ZA | ZA |
| Sapindales | Meliaceae | *Melia azedarach* | F |  |  | CA & ZA |
| Sapindales | Meliaceae | *Swietenia chickrassa* | A |  |  | CA |
| Sapindales | Rutaceae | *Calodendrum capense* | F |  | ZA | ZA |
| Sapindales | Rutaceae | *Citrus limon* | F |  |  | CA & ZA |
| Sapindales | Rutaceae | *Citrus sinensis* | F |  |  | CA & ZA |
| Sapindales | Simaroubaceae | *Ailanthus altissima* |  | NK |  | CA |
| Malvales | Malvaceae | *Bombax ceiba* | A |  |  | CA |
| Malvales | Malvaceae | *Brachychiton acerifolius* | F |  |  | CA |
| Malvales | Malvaceae | *Brachychiton australis* | F |  |  | CA |
| Malvales | Malvaceae | *Brachychiton discolor* | F |  |  | CA & ZA |
| Malvales | Malvaceae | *Brachychiton populneus* |  | NK |  | CA |
| Malvales | Malvaceae | *Brachychiton rupestris* | F |  |  | CA |
| Supplementary Table I. Continued. | | | | | | |
| Order | Family | Species | Non-Competent | Competent | Native Range | Infested Distribution |
| Malvales | Malvaceae | *Ceiba pentandra* | F |  |  | ZA |
| Malvales | Malvaceae | *Ceiba speciosa* | F |  |  | CA |
| Malvales | Malvaceae | *Chiranthodendron pentadactylon* | A |  |  | CA |
| Malvales | Malvaceae | *Dombeya cacuminum* |  | NK |  | CA |
| Malvales | Malvaceae | *Dombeya wallichii* | A |  |  | CA |
| Malvales | Malvaceae | *Firmiana simplex* | F |  |  | CA |
| Malvales | Malvaceae | *Grewia occidentalis* | F |  | ZA | ZA |
| Malvales | Malvaceae | *Heliocarpus sp.* | A |  |  | CA |
| Malvales | Malvaceae | *Luehea divaricata* | F |  |  | CA |
| Malvales | Malvaceae | *Pseudobombax ellipticum* | A |  |  | CA |
| Malvales | Malvaceae | *Tilia americana* | F |  |  | CA |
